# A new location to split Cre recombinase for protein fragment complementation

**DOI:** 10.1111/pbi.12726

**Published:** 2017-04-20

**Authors:** Maryam Rajaee, David W. Ow

**Affiliations:** ^1^ Plant Gene Engineering Center South China Botanical Garden Chinese Academy of Sciences Guangzhou China; ^2^ University of Chinese Academy of Sciences Beijing China

**Keywords:** site‐specific recombination, gene stacking, protein fragment complementation, alpha‐complementation

## Abstract

We have previously described a recombinase‐mediated gene stacking system in which the Cre recombinase is used to remove *lox*‐site flanked DNA no longer needed after each round of Bxb1 integrase‐mediated site‐specific integration. The Cre recombinase can be conveniently introduced by hybridization with a *cre*‐expressing plant. However, maintaining an efficient *cre*‐expressing line over many generations can be a problem, as high production of this DNA‐binding protein might interfere with normal chromosome activities. To counter this selection against high Cre activity, we considered a split‐*cre* approach, in which Cre activity is reconstituted after separate parts of Cre are brought into the same genome by hybridization. To insure that the recombinase‐mediated gene stacking system retains its freedom to operate, we tested for new locations to split Cre into complementing fragments. In this study, we describe testing four new locations for splitting the Cre recombinase for protein fragment complementation and show that the two fragments of Cre split between Lys244 and Asn245 can reconstitute activity that is comparable to that of wild‐type Cre.

## Introduction

**Figure 1:**
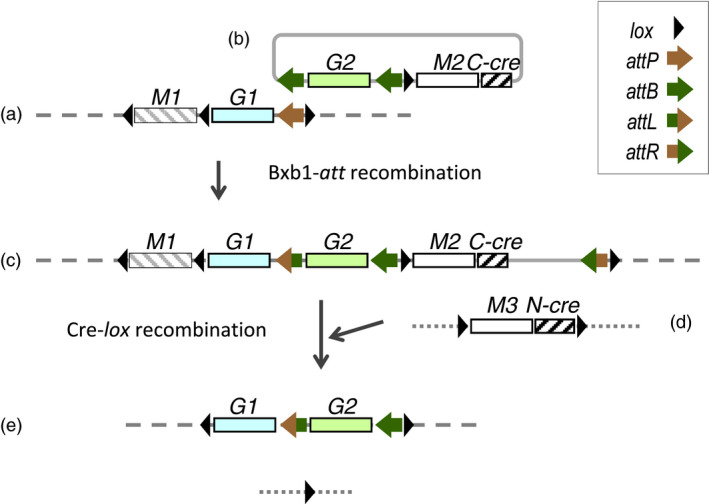
Reconstitution of Cre activity for removing DNA no longer needed after site‐specific integration. Scheme for recombinase‐mediated gene stacking shows the chromosomal target construct with an *attP* site (a) recombining with an integrating vector (b) that also includes a C‐Cre peptide encoding gene. After site‐specific integration from *attP* recombination with the *M2* distal *attB* site to yield the configuration shown in (c), the construct encoding N‐Cre peptide (d) is introduced by genetic hybridization to yield the expected configuration shown in (e). *M1, M2 and M3* represent marker genes, and *G1 and G2* represent trait genes. Symbols of recombination sites defined in legend. Note that the *M3‐N‐cre* fragment is also excised in the F1 (e).

A long‐term aim of this laboratory has been to develop site‐specific gene stacking to ease the introgression of transgenes from transformable lines to elite field cultivars (Hou *et al*., 2014). Each step of the reiterative gene stacking scheme involves using the Bxb1 integrase to direct the insertion of new DNA into a genomic Bxb1 attachment site (*attP* or *attB* site), followed by introduction of the 343aa Cre recombinase to delete the 34 bp *lox*‐site flanked DNA no longer needed after site‐specific integration. While the Bxb1 integrase can be transiently introduced along with the integrating DNA, the Cre recombinase is most conveniently introduced from hybridization to a *cre*‐expression line. Maintaining an efficient *cre*‐expressing line over many generations, however, could potentially be a problem as high expression of *cre* in petunia and tomato has been associated with crinkled leaves and/or reduced fertility (Que *et al*., 1998; Cappoolse *et al*., 2003). As recombinases are DNA‐binding proteins, it remains possible that high production of these proteins could interfere with normal chromosome activities. To insure against this possibility, we considered a split‐*cre* approach as shown in Figure 1, in which the integrating vector brings along a portion of *cre*, and after integration, the rest of *cre* can be subsequently introduced from hybridization.

**Figure 2:**
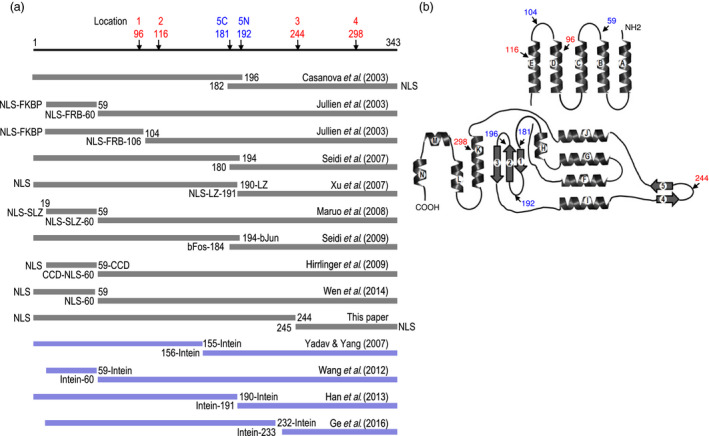
Linear depiction (a) and X‐ray crystal structure (b) of Cre recombinase. (a) Split‐Cre fragments that can reconstitute Cre activity through protein fragment complementation shown with grey bars. Split‐Cre fragments for reconstruction of whole Cre protein through protein splicing shown with blue bars. Numbers refer to the aa residues. Fusion to the following: NLS = nuclear localization sequence; LZ = leucine zipper; SLZ = synthetic leucine zipper; bJun and bFos = AP‐1 transcription regulatory proteins; CCD = coiled coil domain of the yeast transcriptional activator GCN4; FKBP12 = FK506 binding protein; FRB = binding domain of the FKBP12‐rapamycin‐associated protein. Intein= from *Cyanobacterium synechocystis* Ssp DnaE. ATG added to N‐terminus of all C‐Cre fragments shown. (b) X‐ray crystal structure from Guo *et al*. (1997) showing the 14 alpha‐helical segments (A‐N) and five beta‐sheets (1–5). Arrows point to relevant aa residues. Blue lettering, previously tested locations; red lettering, the four new locations tested in this study.

Figure 2a lists the various reports on splitting Cre into two peptides. The first approach is based on protein fragment complementation, where Cre activity is reconstituted from separate peptides. Casanova *et al*. (2003) first reported testing various pairs of nonfunctional N‐terminal and C‐terminal Cre fragments at break points between aa160 and aa203 and found one pair, aa1‐196/aa182‐343, that reconstituted activity in a transient assay in CV1‐5B monkey cells, but at only 33% efficiency compared to wild‐type Cre. Seidi *et al*. (2007) also reported success with a split‐Cre pair at the same region, aa1‐194 and aa180‐343, and with up to 68% excision efficiency in a transient assay in COS‐7 monkey cells. More recently, Wen *et al*. (2014) reconstructed Cre from the pair aa1‐59/aa60‐343 with and without the SV40 NLS and in an excision assay of *A. rhizogene*‐mediated transformation of tobacco root hair, up to 67% of wild‐type Cre efficiency was reported.

Uses of heterodimerizing peptides to facilitate the reassembly of split‐Cre complementating peptides have also been tested. Jullien *et al*. (2003) reported reconstituting the pairs aa19‐59/aa60‐343 and aa19‐104/106‐343, in which each N‐terminal fragment (N‐fragment) was fused to FKBP12 (FK506 binding protein) while each C‐terminal fragment (C‐fragment) was fused to FRB (binding domain of the FKBP12‐rapamycin‐associated protein). Upon addition of rapamycin that induces FKBP12/FRB interaction, Cre activity was reconstituted in both a transient and a stable excision assay in a Rat2/CALNLZ cell line. In the transient assay, the aa19‐104/106‐343 pair showed higher recombination activity, whereas in the stable excision assay, the aa19‐59/aa60‐343 pair was more efficient. In a study by Xu *et al*. (2007), each N‐ and C‐fragment of the aa1‐190/aa191‐343 pair was fused to antiparallel leucine zippers. In transient and transgenic mouse cells, excision efficiencies were about 30% compared to wild‐type Cre. Maruo *et al*. (2008) tested parallel binding modules and reported that a Zip(+)/(−) synthetic leucine zipper based on the vitellogenin‐binding protein from a chicken b‐ZIP family was most efficient among three heterodimerizing peptide pairs of aa19‐59/aa60‐343 tested in Cos‐7 monkey cells and in immature mouse neurons, but its efficiency was not compared to wild‐type Cre. Seidi *et al*. (2009) used the AP‐1 transcription regulatory proteins bJun and bFos in a transient monkey COS7 cell assay that showed bJun‐Cre (aa‐1‐194) and bFos‐Cre (aa‐184‐343) yielded 23% deletion efficiency compared to wild‐type Cre. Finally, Hirrlinger *et al*. (2009) reported using the coiled coil domain of the yeast transcriptional activator GCN4 on the split‐Cre pair aa19‐59/aa60‐343 in transgenic mice and found 26% deletion efficiency.

In contrast to reconstituting activity from protein fragment complementation, split inteins can reconstruct a whole Cre protein by splicing together separate protein peptides. The first report of using split inteins to reconstruct Cre was described in a patent issued to Dupont (Yadav and Yang, 2007). The split inteins from *Cyanobacterium synechocystis DnaE* reconstructed Cre from the pair of aa1‐155/aa156‐343 fragments. Using this same approach, Wang *et al*. (2012) reconstructed Cre peptides aa19‐59 and aa60‐343 and reported high Cre activity in mouse brain tissue. Han *et al*. (2013) tested the same *Ssp DnaE* split intein on the Cre pair aa1‐190/aa191‐343 and obtained excision activity in tobacco. Based on the number of leaf explants resistant to the herbicide Basta, up to 77% of wild‐type Cre efficiency was report. More recently, Ge *et al*. (2016) tested five Cre pairs (aa1‐128/aa129‐343, aa1‐153/aa154‐343, aa1‐190/aa191‐343, aa1‐232/aa233‐343 and aa1‐281/aa282‐343) in a transient tobacco explant assay and concluded that the best pair for split‐intein reconstruction of Cre was aa1‐232/aa233‐343. In transgenic *Arabidopsis*, this split‐intein reconstruction of Cre was reported to be near 100% efficiency in F1 hybrids.

In terms of location flexibility to split Cre, as well as restoration of Cre activity, the split‐intein approach to reconstruct a whole Cre protein would seem preferable to complementation activity with separated peptides. However, as an important aim for developing our ‘open‐source’ gene stacking system was to permit the freedom to operate for commercial crop improvement (Ow, 2016), we had to consider the intellectual property constraints of the Dupont intein‐Cre patent that is in effect until 2023 (Yadav and Yang, 2007). With the Cre fragment complementation approach, there have been two patent applications filed based on the work of Jullien *et al*. (2003) and Xu *et al*. (2007) that claim the use of specific heterodimerizing peptides along with specified split‐Cre locations, namely after aa 59, 104 and 190 (Gu and Xu, 2009; Herman and Jullien, 2004). As issued patents of these were not found, it seems likely that these applications have been abandoned. Surprisingly, however, despite prior art on splitting Cre after aa 59, a Chinese patent was issued recently based on the work of Wen *et al*. (2014) that claimed splitting Cre after aa 59 (Gao *et al*., 2015). Given the freedom‐to‐operate uncertainty, we thought it would be preferably to create our own version of Cre fragment complementation.

As indicated in Figure 2a, previous reports have led to relatively few break points that can yield Cre fragment complementation, and they are all within loops that connect alpha‐helical segments or beta‐sheets. Four Cre pairs were split at the region between aa180‐196 within loops linking beta‐sheets 1, 2 and 3; another four pairs split after aa59 between alpha‐helixes B and C, and a single report of splitting at aa104‐106 between alpha‐helixes D and E (Figure 2b). Likewise, we thought it might be possible to split the protein within the loop connecting beta‐sheets 4 and 5. Additionally, because it was convenient to generate C‐fragments that begin with ATG start codon, we also included splitting Cre after Asn96, Val116 and Asp298 (Figure 2b), even though each of them disrupts an alpha‐helical segment. Here, we report that the break point between Lys244 and Asn245 indeed yields two peptides that can undergo Cre fragment complementation activity in *E. coli* and in transgenic *Arabidopsis*. In *Arabidopsis,* the recombination efficiency by hybrid‐reconstituted Cre was comparable to that of wild‐type Cre and was obtained without the need of heterodimerizing peptides.

## Results

### Testing new split‐Cre pairs for protein fragment complementation in *E. coli*

**Figure 3:**
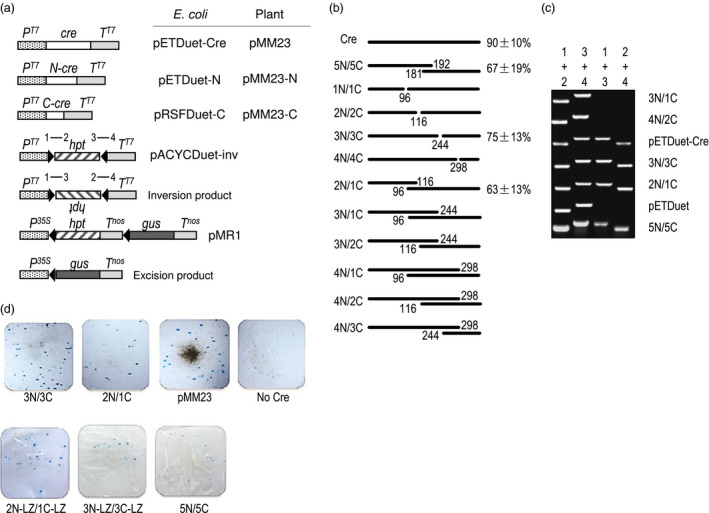
Reconstitution of Cre activity among split‐Cre pairs in bacterial and plant cells. (a) Not to scale depiction of DNA constructs used for *E. coli* and plant cells, only relevant DNA segments shown. *N*‐*cre* and *C‐cre* encode N‐ and C‐terminal fragments of Cre, respectively. *P* ^*T7*^ = T7 phage promoter; *P* ^*35S*^ = CaMV 35S RNA promoter; *T* ^*T7*^ = T7 phage transcription terminator; *T* ^*nos*^ = *nos* terminator; *hpt* = hygromycin phosphotransferase gene; *gus*=beta‐glucuronidase gene; numbers 1–4 refer to PCR primers. Genes transcribed left to right except for *hpt* in the inversion product where upside‐down lettering indicates transcription from right to left. (b) Depiction of 10 split‐Cre pairs tested along with wild‐type Cre and the 5N/5C positive control. Negative control not shown. % = % of *E. coli* colonies found to show Cre‐mediated inversion (mean ± SD of three independent experiments; 16 colonies tested per experiments). 0% not shown. (c) Representative PCR analysis of the presence of the 1 + 3 and 2 + 4 PCR products indicative of site‐specific inversion, 3N/1C and 4N/2C are representative pairs that failed to show Cre activity. (d) GUS histochemical staining of bombarded onion epidermis. Blue spots show GUS activity to indicate formation of excision product from site‐specific excision of *hpt‐T* ^*nos*^ blocking DNA . LZ = leucine zipper of Max and Myc.

To examine whether the 343 amino acid Cre recombinase can be functionally reconstituted when split at regions other than the three previously reported, we chose to split Cre after Asn96 (Figure 2a, location 1), Val116 (location 2), Lys244 (location 3) and Asp298 (location 4). Locations 3 and 4 are within the C‐terminal end of the protein that have not been previously tested (Figure 2a). Locations 1, 2 and 4 were also chosen because each could be split such that the C‐terminal fragment begins with an ATG start codon, bypassing the need to add another amino acid to the C‐terminal peptide, which was the case for splitting at location 3 (after Lys244). Based on the Cre X‐ray crystal structure (Guo *et al*., 1997) of five alpha‐helical segments (A‐E) connected by short loops in its amino‐terminal domain and nine alpha‐helical segments (F‐N) along with five beta‐sheets (1‐5) in its carboxyl‐terminal domain (Figure 2b), our location 1 cut is located in α‐helix D between Asn96 and Met97 resulting in peptide 1N (1‐96 aa) and 1C (97‐343 aa) for N‐ and C‐terminal moieties, respectively. Location 2 is in α‐helix E between Val116 and Met 117 yielding 2N (1‐116 aa) and 2C (117‐343 aa) fragments. Location 3 cut is between the 4th and 5th beta‐sheets after Lys244 resulting in 3N (1‐244 aa) and 3C (245‐343 aa) fragments, and location 4 is in the K alpha‐helix between Asp298 and Met299 to yield 4N (1‐298 aa) and 4C (299‐343 aa) peptides (Figure 2b). By mixing N‐terminal and C‐terminal fragments of the different pairs, a total of 10 pairs, including six overlapping ones, were tested for Cre fragment complementation in *E. coli* (Figure 3b). For use as a positive control, we also generated a fifth split‐Cre pair of aa1‐192/aa181‐343 because this region has been reported to work by four prior studies (ATG start codon added before aa181; referred to as 5N/5C).

Each N‐terminal and C‐terminal gene fragment was expressed in pETDuet and pRSFDuet vectors to produce the ampicillin‐resistant pETDuet‐N and kanamycin‐resistant pRSFDuet‐C series of constructs, respectively (Figure 3a). A third plasmid that is chloramphenicol resistant, pACYCDuet‐inv served as a reporter for site‐specific recombination. This construct has a hygromycin coding region (*hpt*) flanked by oppositely oriented *lox* sites such that site‐specific recombination inverts the *hpt* fragment to produce a predicted molecular structure detectable by PCR. For a positive control, the full‐length *cre* gene was expressed from pETDuet‐Cre. All three plasmid types (pETDuet, pRSFDuet and pACYCDuet) are compatible for co‐propagation in the *recA1* homologous recombination impaired *E. coli* strain DH5α (Grant *et al*., 1990).

In the absence of site‐specific inversion, PCR products of pACYCDuet‐inv can be detected using primer pairs 1 + 2 and 3 + 4, but not from primer pairs 1 + 3 and 2 + 4 (Figure 3a). If inversion takes place, which is a reversible event, PCR products can also be detected using primer pairs 1 + 3 and 2 + 4. As expected from the controls, 90% of the colonies derived from pACYCDuet‐inv showed site‐specific inversion when cotransformed by pETDuet‐Cre, whereas none was found when cotransformed with the pETDuet empty vector. For the cotransformation of N and C pairs, which requires the co‐introduction of three plasmids instead of two for the controls, the 5N/5C (aa1‐192/aa181‐343) pair indeed showed site‐specific inversion in 67% of the colonies. This confirms earlier reports of reconstitution of Cre activity when split at the region between aa181‐196. For the pairs corresponding to locations 1, 2, 3, 4, only 3N/3C (aa1‐244/aa245‐343) scored positive for reconstituted Cre activity. This may not be surprising as locations 1, 2 and 4 disrupt alpha‐helical segments. However, among the six overlapping pairings of N‐ and C‐fragments, reconstituted Cre activity was found in 2N/1C (aa1‐116/aa96‐343). It is interesting to note that 2N includes alpha‐helix D, while 1C includes alpha‐helix E, and the overlap provides a full set of alpha‐helical segments. However, the same logic does not hold for the other overlapping pairs, 3N/1C, 3N/2C, 4N/1C, 4N/2C and 4N/3C. Given the positive results of 3N/3C and 2N/1C, we advanced them to the next step of testing for Cre fragment complementation in plant cells.

### Reconstitution of Cre activity in plant transient assays

For scoring site‐specific recombination in plant cells, a reporter construct was initially used in which the *gus* (β‐glucuronidase) coding region is prevented from expression by an upstream blocking DNA that is itself flanked by a set of directly oriented *lox* sites (pMR1, Figure 3a). The *hpt* coding region along with a *nos* terminator (*polyA*) region was used as the blocking DNA. Cre‐mediated excision of the blocking DNA permits *gus* expression from the CaMV 35S RNA promoter (*P* ^*35S*^) in one of the excision products, and β‐glucuronidase activity can be visually detected by blue staining. N‐ and C‐fragments, and the full‐length *cre* gene, were also expressed in separate constructs transcribed by *P* ^*35S*^ (pMM23 series constructs, Figure 3a). Microparticle bombardment (biolistics) was used to deliver the N‐ and C‐fragment‐expressing constructs along with the reporter construct into onion epidermal cells. Blue spots were visible 12 hours after bombardment with both the 3N/3C and 2N/1C pairs, with 3N/3C nearly as efficient for recombination as the wild‐type Cre control. Surprisingly, our positive split‐Cre control pair 5N/5C was less effective. To examine whether heterodimerizing peptides could further enhance their reconstitution of activity, leucine zippers of transcription factors were added to both 3N/3C and 2N/1C, with *Myc* to the C‐terminus of the N‐Cre fragment and *Max* to the N‐terminus of the C‐Cre fragment. However, neither leucine zipper‐containing pairs were found to be as effective as the nonleucine zipper progenitors.

**Figure 4:**
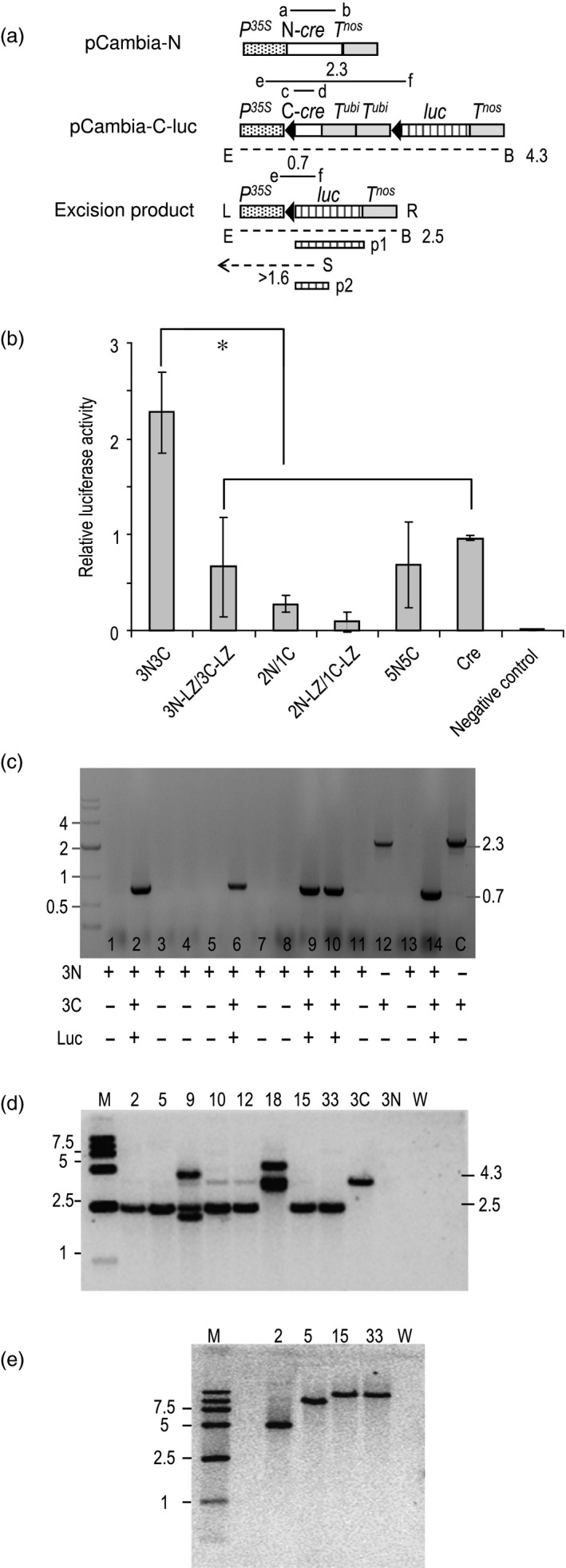
Reconstitution of Cre activity for recombination of plasmid and chromosomal DNA. (a) Not to scale depiction of constructs used, only relevant DNA segments shown. Genetic elements as in Figure 3(a). *P* ^*35S*^ = CaMV 35S RNA promoter for plants; *T* ^*nos*^ = *nos* terminator; *T* ^*ubi*^ = ubiquitin gene terminator*; luc* = firefly luciferase gene; a to f = primers used. Dashed lines indicate extent of DNA hybridizing to probes p1 and p2 when cleaved with *Eco*RI (E) plus *Bst*EII (B) or with *Sph*I (S). Numbers refer to length of DNA size in kb. (b) Transient expression in *Arabidopsis* protoplasts. Values are mean ± SD; *n* = 9. *P < 0.05, Dunnett's *t*‐test. (c) Representative PCR detection of deletion of chromosomal copy of *lox*‐flanked DNA that indicates reconstitution of activity from 3N/3C split‐Cre pair. Summary of detection of 3N‐ and 3C‐specific PCR products and luciferase activity shown below gel. Primers a+b detects N‐fragment, c + d detects C‐fragment, e + f detects excision junction. Leftmost lane shows size markers in kb. (d) Southern blot of eight F1 plant lines. DNA cleaved with *Eco*RI (E) plus *Bst*EII (B) and hybridized to probe p1. M = size markers in kb. 3C = pCambia‐3C‐luc line, 3N = pCambia‐3N line, W = wild‐type control. (e) Southern blot of F1 plant DNA cleaved with *Sph*I (S) and hybridized to probe p2. M = size markers in kb. W = wild‐type control.

Although the visual assay concluded that the 3N/3C and 2N/1C pairs work, a second test was conducted in *Arabidopsis* protoplasts to assess their relative recombination proficiency. Several modifications were made to the excision assay. First, for ease in quantitation, we switched to using *luc* (firefly luciferase gene) as a reporter. Second, in preparation of further testing in transgenic plants in which recombination activity in the nucleus is scored, an SV40 NLS was added to the N‐terminus of the N‐fragment and to the C‐terminus of the C‐fragment. Third, to reduce the cotransfer of constructs from 3 to 2, the corresponding *C‐cre* fragment of each pair followed by two rice *ubi1* terminators (*T* ^*ubi1*^) was used as the blocking DNA (pCambia‐C‐luc, Figure 4a). For the control experiment with the wild‐type Cre, the *luc* reporter construct uses as the blocking DNA a *bar* (basta resistance) gene instead of *C‐cre* (construct not shown).

Twenty hours after polyethylene glycol (PEG)‐mediated uptake of DNA into *Arabidopsis* protoplasts, the cells were assayed for *luc* expression as an indication of site‐specific excision of the blocking DNA. Compared to wild‐type Cre, 2N/1C and 5N/5C showed 27% and 69% activity, respectively. However, 3N/3C was about 2.5‐fold more active than wild‐type Cre (Figure 4b). As in the β‐glucuronidase assay in onion cells, the leucine zipper additions to 3N/3C and 2N/1C reduced rather than increased recombination.

### Cre‐fragment complementation in transgenic *Arabidopsis*

Having shown that the NLS‐linked 3N/3C pair is the most active in the two transient assays, we sought to test for recombination of chromosomal DNA. The constructs pCambia‐3N and pCambia‐3C‐luc were separately transformed into *Arabidopsis*. Putative single‐copy lines were screened in T1 plants by qPCR, followed by testing for a 3:1 segregation of the transgene among T2 seedlings. These two assays narrowed the number of putative single‐copy lines to 6 for pCambia‐3N and 12 for pCambia‐3C‐luc. From these 18 lines, a total of 27 crosses were conducted between pCambia‐3N and pCambia‐3C‐luc plants. The F1 plants from these crosses were PCR genotyped to determine the plants that harboured deletion of the *C‐cre‐T* ^*ubi1*^ *‐T* ^*ubi1*^ fragment, which would fuse *P* ^*35S*^ to *luc*. PCR primer pair e+f (Figure 4a) should yield either a 2.3‐kb product prior to excision or a 0.7‐kb band after excision. As shown in Figure 4c, detection of the 0.7‐kb PCR product was found in a representative sampling of F1 plants. Moreover, this correlated with expression of *luc*. All 27 independent crosses yielded progeny that showed reconstitution of Cre activity as defined by the PCR and luciferase assays. Both pCambia‐3N and pCambia‐3C‐luc constructs, as determined by PCR pairs a + b and c + d, respectively, were detected in the F1 plants that showed excision. In contrast, plants that failed to show excision lacked either pCambia‐3N or pCambia‐3C‐luc DNA in their genomes (Figure 4c).

Ten representative recombination‐junction PCR products from the progenies of the 27 independent crosses were sequenced, and all showed precise site‐specific recombination. Additionally, Southern blots were conducted on the F1 and F1 backcrossed (BC1) plants to rule out the possibility that the PCR data had arisen from PCR‐mediated recombination of templates with a common *lox* site. *Eco*RI and *Bst*EII cleave just inside the border of the transgene and should yield a *luc*‐hybridization band of ~4.3 kb before recombination and ~2.5 kb after recombination (Figure 4a). As shown in a Southern blot (Figure 4d) on plants previously found by PCR to have undergone excision, some lines (lines 9, 18) show multiple hybridizing bands to the *luc* probe p1. This suggests that their genomes harbour other imperfectly integrated T‐DNA copies (Figure 4d). In some other lines (lines 10, 12), a faint before‐excision band (~4.3 kb) was also detected in addition to the excision‐specific product, suggesting that the excision was not complete. However, in the remaining four lines (lines 2, 5, 15 and 33), only a single ~2.5‐kb excision‐specific band was detected to indicate a homogenous recombination event. To rule out the possibility that the internal *Eco*RI‐*Bst*EII fragment may represent the excision of more than a single pCambia‐C‐luc derived T‐DNA, the genomic DNA of lines 2, 5, 15 and 33 were cleaved with *Sph*I and hybridized to *luc* probe p2. As *Sph*I cleaves only once within the T‐DNA, p2 should detect the T‐DNA left border band. As shown in Figure 4e, a single border band >1.6 kb was indeed detected in these four lines, confirming that each line harbours only a single T‐DNA copy. However, it was surprising to find that lines 15 and 33 each showed a band of approximate the same size, thereby raising the possibility that they may be clonal due to a mix‐up of the seeds. If that were the case, then we can only state that three of seven lines (rather than four of eight lines) examined showed efficient deletion of a single‐copy T‐DNA.

### Testing transmission of the excision event

Given that the data show efficient recombination in somatic cells, we sought to test whether the recombination event could transmit to the next generation. For the F1 plants that showed excision, the N‐fragment locus (*3N*) and C‐fragment locus (*3C*) should be hemizygous (*NnCc*, lower case lettering indicating the absence of transgene) and four gamete types should be possible: *NC*,* Nc*,* nC* and *nc*. In an outcross to wild‐type (*nc*), this would yield progeny with the following genotypes: *NnCc*,* Nncc*,* nnCc* and *nncc*. If recombination took place in germ‐line cells, then we would expect to recover plants with the *nnCc* genotype but with an excision event at the *C* locus. Progenies from 16 of 27 F1 to wild‐type backcrosses were randomly chosen for PCR detection of the excision event (~0.7 kb e + f PCR product) as well as for the *3N* and *3C* fragments (a + b and c + d PCR products, respectively). However, among 50 progeny from each backcross that were positive for the excision event, all harboured the *3N* locus (data not shown). Hence, we conclude that the plants that harbour the excision configuration are genotypically *NnCc* and that the co‐assortment of both the *3N* and *3C* loci permitted another round of reconstituted Cre‐mediated site‐specific recombination in the somatic cells of the backcross progenies.

## Discussion

In our recombinase‐mediated gene stacking scheme, Cre is used to delete DNA no longer needed after each site‐specific integrations step, such as selectable markers and plasmid backbones. Cre‐mediated excision of *lox*‐flanked DNA is generally efficient, but depends on the *cre*‐expression donor line used in the genetic cross. In recent work, we found that a *cre* line used previously was less efficient than we had expected, resulting in all F1 plants chimeric with a mixture of cells with or without recombination (Hou *et al*., 2014). We had suspected that the *cre* line might have become less effective over time. Although gene silencing over the generations could have occurred with any transgene, there is some reason to suspect that high expression of a DNA‐binding recombinase could interfere with normal chromosome activities. Hence, we sought to create *cre* lines with controllable *cre* DNA activity. Chemical induction (Joubes *et al*., 2004; Zuo *et al*., 2001) and heat induction of *cre* expression have been described (Cuellar *et al*., 2006; Hoff *et al*., 2001; Khattri *et al*., 2011; Wang *et al*., 2005; Zhang *et al*., 2003). However, we favour using a split‐*cre* system as it should be possible, as illustrated in Figure 1d, to flank the *M3‐N‐cre* DNA with directly oriented *lox* sites. With an inducible *cre* system, keeping a *cre* line over the generations might be difficult, as any leaky expression of *cre* would excise itself.

What prompted us to find a new split‐Cre pair instead of using those described in the literature is due to our desire to keep the recombinase‐mediated gene stacking system as an open‐source system with freedom to operate (Chen and Ow, 2017; Ow, 2016). The split‐intein patent (Yadav and Yang, 2007) has broad claims that are difficult to invent around. For the Cre fragment complementation approach, at least three applications were found that claim specific locations by which Cre can be split into two (Herman and Jullien, 2004; filed 2002; Gu and Xu, 2009; filed 2009; Gao *et al*., 2015; filed 2013). At least one of these applications has since been issued (Gao *et al*., 2015).

Under these circumstances, we undertook the task to a search for a new location for Cre fragment complementation. The positive outcome from this research is that we indeed found a new split‐Cre pair that can reconstitute activity that is comparable to the wild‐type Cre control. Unlike other studies that relied exclusively on scoring for recombination through reporter gene expression, or by PCR, both of which cannot detect the percentage of substrates that have not undergone recombination, we followed up our initial analysis with a Southern analysis which showed that from eight randomly selected progenies, the excision‐specific hybridizing band was found in seven of them, and with only faint or undetectable hybridization for the band representing the lack of excision. This shows that the Cre pair brought together by hybridization was highly effective at the F1 generation.

Yet, despite the high efficiency of recombination in somatic cells, the one surprising outcome of this study was that backcrossed progenies that showed excision invariably harboured both the *N‐cre* and *C‐cre* loci. This suggests that the excision events were generated *de novo* from the reconstitution of *N‐cre* and *C‐cre* in the backcrossed progeny generation. Both *N‐cre* and *C‐cre* genes were transcribed by the CaMV 35S RNA promoter and this promoter has previously been used to express *cre* to cause excision that can be transmitted through the germ‐line, although the efficiency varies depending on the particular *cre* donor line. It remains possible that a lower level of expression was caused by having both *N‐cre* and *C‐cre* transcribed by the same promoter. Whatever the reason, it remains a next engineering challenge to test different promoters, including germ‐line‐specific promoters for more effective transmission of the recombination event (Li *et al*., 2007; Mlynarova *et al*., 2006; Van Ex *et al*., 2009; Verweire *et al*., 2007). As we have now progressed to implementing the recombinase‐mediated gene stacking system in rice (Li *et al*., 2016), future testing of these germ‐line‐specific promoters will be conducted on this crop.

## Experimental procedure

### DNA constructs

Standard recombinant DNA methods were used throughout (Sambrook and Russell, 2001). Primer and DNA linker sequences shown in Table S1.

pETDuet‐Cre: The *cre* gene was PCR amplified (Biolabs Phusion^®^ High‐Fidelity DNA Polymerase) from pMM23 (Qin *et al*., 1994) using primers N‐F and C‐R and inserted between *Eco*RI and *Kpn*I restriction sites of pETDuet (pETDuet™‐1, Novagen).

pETDuet‐N series constructs: 1*N*‐*cre* fragment was PCR amplified from pMM23 using primers N‐F and 1N‐R, 2*N*‐*cre* used primers N‐F and 2N‐R, 3*N*‐*cre* with primers N‐F and 3N‐R, 4*N*‐*cre* with primers N‐F and 4N‐R*,* and 5*N*‐*cre* with primers N‐F and 5N‐R. Each of the *N*‐*cre* DNA was inserted between *Eco*RI and *Avr*II restriction sites of pETDuet (pETDuet™‐1, Novagen).

pRSFDuet‐C series constructs: 1*C‐cre* fragment was PCR amplified from pMM23 using primers 1C‐F and C‐R, 2*C*‐*cre* fragment amplified using primers 2C‐F and C‐R, 3*C*‐*cre* fragment with primers 3C‐F and C‐R, 4*C*‐*cre* fragment with primers 4C‐F and C‐R, and 5*C*‐*cre* fragment with 5C‐F and C‐R. Each of the *C*‐*cre* fragments was inserted between *Eco*RI and *Kpn*I restriction sites of pACYCD (pACYCDuet™‐1, Novagen).

pACYCDuet‐in: The *hpt* gene was PCR amplified from pZH36 (ZG Han, Ow lab, unpublished) using primers lhl‐F and lhl‐R with overhanging *lox* sites to create the *lox‐hpt‐*(inverted *lox*), which was then inserted between the *Eco*RI and *Avr*II restriction sites of pACYCD (pACYCDuet™‐1, Novagen).

pMM23‐N series and pMM23‐C series constructs : 2*N‐cre*, 3*N*‐*cre,* 5*N*‐*cre*, 1*C‐cre*, 3*C*‐*cre* and 5*C*‐*cre* fragments were PCR amplified with same or analogous primers for the pETDuet‐N and pETDuet‐C series plasmids but with *Kpn*I and *Sph*I restriction sites (N‐F1, 2N‐R1, 3N‐R1, 5N‐R1 for *N‐cre* fragments and 1C‐F1, 3C‐F1, 5C‐F1 and C‐R1 for *C‐cre* fragments) for inserting between *P* ^*35S*^ (*Kpn*I) and *T* ^*nos*^ (*Sph*I) of pMM23.

pMM23‐NLZ series constructs: 2N‐LZ *cre* and 3N‐LZ‐*cre* fragments were PCR amplified from pMM23 using primers N‐F2 and 2NLZ‐R or 3NLZ‐R for insertion into the intermediate vector pMD18‐T (Takara) in between *Kpn*I and *Xho*I. Afterwards, a *Myc* (Ayer and Eisenman, 1993) linker (Beijing AUGCT Biotechnology Co., Ltd.) was inserted between the *Xho*I site and the vector‐derived *Sph*I site of pMD‐18T (C‐terminus of the *N‐Cre* fragment) to make 2NLZ and 3NLZ. Finally, 2NLZ and 3NLZ fragments were retrieved by cleavage with *Kpn*I and *Sph*I and inserted between *P* ^*35S*^ (*Kpn*I) and *T* ^*nos*^ (*Sph*I) of pMM23.

pMM23‐CLZ series constructs: 1C‐LZ‐*cre* and 3C‐LZ‐*cre* fragments were PCR amplified from pMM23 using primers C‐R2, 1CLZ‐F or 3CLZ‐F for insertion between the *Eco*RI and *Sph*I sites of pMD18‐T (Takara). Afterwards, a *Max* (Ayer and Eisenman, 1993) linker (Beijing AUGCT Biotechnology Co., Ltd.) was inserted between the *Kpn*I and *Eco*RI sites of pMD‐18T (N‐terminus of the *C‐cre* fragment) to make 1CLZ and 3CLZ. Finally, 1C‐LZ‐*cre* and 3C‐LZ‐*cre* fragments were retrieved by cleavage with *Kpn*I and *Sph*I and inserted between *P* ^*35S*^ (*Kpn*I) and *T* ^*nos*^ (*Sph*I) of pMM23.

pMR1: An *Xho*I‐*gus‐Sph*I fragment was PCR amplified from pZH36 (ZG Han, Ow lab, unpublished) using primers Gus‐F and Gus‐R for insertion into a T‐vector (Takara) between the corresponding sites. An *hpt* fragment (with *nos* terminator) was also PCR amplified from pZH36, using primers lhl‐F and lhl‐R with overhanging *lox* sites to create a *Kpn*I‐*lox‐hpt‐lox‐Xho*I fragment for insertion upstream of the *gus* gene between . The *lox‐hpt‐lox‐gus* fragment was then retrieved by cleavage with *Kpn*I and *Sph*I for insertion between *P* ^*35S*^ (*Kpn*I) and *T* ^*nos*^ (*Sph*I) of pMM23.

pCambia‐N series constructs: 2*N‐cre*, 3*N*‐*cre*, 5*N*‐*cre*, were PCR amplified from pMM23 with Nnls‐F and 2N*‐*R2, 3N‐R2 or 5N‐R2 primers (Nnls‐F included a Kozak sequence, ATG and a SV40 NLS). 2N‐LZ and 3N‐LZ fragments were PCR amplified from pMM23‐NLZ series constructs with Nnls‐F and Nlz‐R. Each fragment was inserted between *P* ^*35*^ (*Bgl*II) and *T* ^*nos*^ (*Bst*EII) of pCAMBIA1301 (http://www.cambia.org).

pCambia‐C‐luc series constructs: *C*‐*cre* fragments were PCR amplified from the pMM23‐C series constructs with primers C‐R3 and either 1Cnls‐F, 3Cnls‐F or CLZnls‐F that incorporated a *Kpn*I site, a Kozak sequence, *lox*, ATG and a SV40 NLS to form a *Kpn*I‐*lox*‐*C‐cre*‐*Nde*I fragment into pMD‐18T (Takara). A first *T* ^*ubi1*^ fragment was PCR amplified from pZH36 using primers T^ubi1^‐F with and T^ubi1^‐R and inserted downstream of *lox‐C‐cre* between *Nde*I and *Pst*I sites. A second *T* ^*ubi1*^ fragment was created by PCR primers T^ubi1^‐F2 and T^ubi1^‐lox‐R to create a *Pst*I‐*T* ^*ubi1*^‐*lox‐Bgl*II fragment to insert behind the first *T* ^*ubi1*^ to create *lox*‐*C*‐*cre*‐*T* ^*ubi1*^ *T* ^*ubi1*^ *lox* linkage within pMD‐18T, upon which the *Kpn*I‐*Bgl*II fragment was transferred to pCambia1301. Finally, a *Bgl*II‐*luc‐Bst*EII fragment amplified by primers luc‐F and luc‐R from pYWP72 (Yau *et al*., 2011) was inserted in place of the *gus* gene in pCambia1301.

pCambia‐bar‐luc: a *Kpn*I‐*lox*‐*bar‐Nde*I fragment was PCR amplified from pZH210B (ZG Han, Ow lab, unpublished) using primers Bar‐F and Bar‐R to replace the *Kpn*I‐*lox‐C*‐*cre‐Nde*I fragment in pCambia‐C‐luc.

pCambia‐Cre: A *Bgl*II‐c*re‐Bst*EII fragment was PCR amplified from pMM23 using Nnls‐F and C‐R4 to insert between *P* ^*35S*^ (*Bgl*II) and *T* ^*nos*^ (*BstE*II) of pCAMBIA1301.

### *E. coli* assay

*E. coli* DH5α (*F*‐ *endA1 glnV44 thi‐1 recA1 relA1 gyrA96 deoR nupG Φ80dlacZΔM15 Δ(lacZYA‐argF)U169 hsdR17(r*_*K*_^*−*^ *m*_*K*_^*+*^ *), λ–*) (Grant *et al*., 1990) was used throughout for recovery of recombinant molecules. For the experiment shown in Figure 3b, 100 μL competent cells were transformed with 100 ng of each plasmid construct, and resistant colonies were scored on plates containing 34 mg/L chloramphenicol, 100 mg/L ampicillin and if needed 50 mg/L kanamycin.

### Transient expression in onion epidermal cells

DNA was purified using Tiangene^®^ High Pure Maxi Plasmid Kit. Onion inner epidermis was placed onto MS medium plates (supplemented by 200 μm of D‐sorbitol and 200 μm D‐mannitol) and subjected to microparticle bombardment as described (Altpeter *et al*., 1996). Afterwards, the plates were incubated in the dark for 14–20 h and the onion epidermis stained for the Gus activity (Jefferson *et al*., 1987).

### Transient expression in *Arabidopsis* protoplasts

Protoplasts were isolated from 10 to 20 leaves of 3‐ to 4‐week‐old *A. thaliana* (cv. Columbia) plants before flowering and transformed by PEG as described (Yoo *et al*., 2007), using for each sample ~1.5 × 10^5^ protoplasts (200 μL volume) and 10 μg of each DNA construct (Tiangene^®^ purified). Luciferase activity was assayed by the DualLuciferase Reporter Assay System (Promega) using a GloMax Multi JR detection Luminometer (Promega) on overnight resting protoplasts and normalized to total protein (Bradford assay kit, Thermo Scientific^®^).

### Transgenic *Arabidopsis*

The constructs shown in Figure 4a were introduced into *Agrobacterium* strain *GV3101* through floral‐dip transformation of *A. thaliana* (cv. Columbia) plants as described (Clough and Bent, 1998). T1 hygromycin‐resistant transformants were selected on an MS medium with 40 μg/L hygromycin. For *N‐cre* plants that lacked a cotransformed reporter gene, *N‐cre* expression was checked by quantitative real‐time PCR using F: 5′ ATTGGCAGAACGAAAACGCT 3′ and R: 5′‐ATCAGCTACACCAGAGACGG‐3′ primers. Total RNA was extracted using Hipure plant RNA mini kit (MAGEN). Reverse transcription was conducted using a PrimeScript™ RT reagent Kit with gDNA Eraser (TAKARA).

### Copy number estimation by quantitative real‐time PCR

Total DNA was extracted using HiPure Plant DNA Midi Kit (Magen, D3162) according to the manufacturer's recommendation. DNA was diluted to 100 μg/μL by ddH_2_O, and 3 μL was used for PCR amplification. Quantitative RT‐PCR reactions were performed in 384‐well blocks using the Go Taq^®^ qPCR Master Mix (Promega, A6001). The *hpt* gene was measured against *AtOXS1* used as an internal positive single‐copy gene control. Primers use for *hpt*: 5′‐TCGTCCATCACAGTTTGCC‐3′ and 5′‐TCGGTCAATACACTACATGGC‐3′; for AtOXS1: 5′‐ACTGTGTCAGATAACCTGCCCGTTG‐3′ and 5′‐GGTTTCTCAGACTTGAGCCTT GGAA‐3′. PCR reactions were 28 cycles, 95 °C 30 s, 60 °C 30 s and 72 °C 30 s. Relative quantification for copy number of T‐DNA insertion was calculated by dividing the Cp values of *hpt* to Cp values of *AtOxs1*.

### Southern hybridization

Southern blots were performed as described previously (Hou *et al*., 2014). DNA was further purified through NucleoBond AX100 columns (Genopure plasmid MiDi kit, Roche, Germany) and concentration determined by a 2000c spectrophotometer (Thermo scientific, Wilmington). ^32^P‐probed membranes were exposed to a phosphor screen for 24 h and detected with Typhoon FLA 9500 (IP: 635 nm, PMT: 500 V, Pixel size 200 μm).

## Supporting information


**Table S1**. Primer sequences.
