# Supplementary material for: A new location to split Cre recombinase for protein fragment complementation
Source: Plant Biotechnol J. 2017 Apr 20;15(11):1420–8. doi: 10.1111/pbi.12726 (PMC5633763; doi:10.1111/pbi.12726)
Supplement: Supplementary file 1 — Table S1. Primer sequences. [file PBI-15-1420-s001.docx]

**Table S1**. Primer sequences.

| Construct | Primer | Primer sequence |
| --- | --- | --- |
| pETDuet-Cre | N-F | 5’-CCCCGAATTCATGTCCAATTTACTGACCGTACAC-3’ (*Eco*RI) |
|  | C-R | 5’-AAAAGGTACCCTAATCGCCATCTTC-3’ (*Kp*nI) |
| pETDuet-N series constructs | N-F | 5’-CCCCGAATTCATGTCCAATTTACTGACCGTACAC-3’ (*Eco*RI) |
|  | 1N-R | 5’-GCGCTTCCTAGGTGTTTAGCTGGCCCAA-3’ (*Avr*II) |
|  | 2N-R | 5’-CACATTCCTAGGTAACCAGTGAAACAGC-3’ (*Avr*II) |
|  | 3N-R | 5’-GCGCTTCCTAGGATTTTCTGACCCGGCAA-3’ (*Avr*II) |
|  | 4N-R | 5’-AAATTCCTAGGAATCTCGCGCGGCTCCGACACGGGC-3’ (*Avr*II) |
|  | 5N-R | 5’-AAATTCCTAGGCTACCCACCGTCAGTACGTGAGATATC-3’ (*Avr*II) |
| pRSFDuet-C series constructs | C-R | 5’-AAAAGGTACCCTAATCGCCATCTTC-3’ (*Kpn*I) |
|  | 1C-F | 5’-GCGCGAATTCATGCTTCATCGTCGGTCC-3’ (*Eco*RI) |
|  | 2C-F | 5’-AAAAGAATTCATGCGGCGGATCCGA-3’ (*Eco*RI) |
|  | 3C-F | 5’-AAAAGAATTCATGGCCCGCGCT-3’ (*Eco*RI) |
|  | 4C-F | 5’- GCGCGAATTCATGGTTAATGGTGTT-3’ (*Eco*RI) |
|  | 5C-F | 5’-GCGCGAATTCATGATCAGGGTTAAA-3’ (*Eco*RI) |
| pACYCDuet-in | lhl-F | 5’-CCCCGAATTCATAACTTCGTATAGCATACATTAT  ACGAAGTTATATGAAAAAGCCTGAACTCACC-3’ (*Eco*RI, *lox*) |
|  | lhl-R | 5’-CCCCCCTAGGATAACTTCGTATAGCATACATTAT  ACGAAGTTATCTATTTCTTTGCCCTCGGACGA-3’ (*Avr*II, *lox*) |
| pMM23-N series | N-F1 | 5’-AAAAGGTACCATGTCCAATTTACTGACCGTACAC-3’ (*Kpn*I) |
|  | 2N-R1 | 5’-AAAAGCATGCTAACCAGTGAAACAGC-3’ (*Sph*I) |
|  | 3N-R1 | 5’-AAAAGCATGCATTTTCTGACCCGGCAA-3’ (*Sph*I) |
|  | 5N-R1 | 5’-AAAAGCATGCCTACCCACCGTCAGTACGTGAGATATC-3’ (*Sph*I) |
| pMM23-C series | 1C-F1 | 5’-AAAAGGTACCATGCTTCATCGTCGGTCC-3’ (*Kpn*I) |
|  | 3C-F1 | 5’-AAAAGGTACCATGGCCCGCGCT-3’ (*Kpn*I) |
|  | 5C-F1 | 5’-AAAAGGTACCATGATCAGGGTTAAA-3’ (*Kpn*I) |
|  | C-R1 | 5’-AAAAGCATGCCTAATCGCCATCTTC-3’ (*Sph*I) |
| pMM23-NLZ series | N-F2 | 5’-CCCCGGTACCATGTCCAATTTACTGACCGTACAC-3’ (*Kpn*I) |
|  | 2NLZ-R | 5’-CACATTCTCGAGTAACCAGTGAAACAGC-3’ (*Xho*I) |
|  | 3NLZ-R | 5’-GCGCTTCTCGAGATTTTCTGACCCGGCAA-3’ (*Xho*I) |
|  | *Myc* linker | 5’-CTCGAGGGGGGGTCTGGGCAAGCAGAGGAGCAAAAGCTCATTTCT  GAAGAGGACTTGTTGCGGAACGACGAGAACAGTTGAAACACAAACTTGAACAGCTACGGAACTCTTGTGCGTGAGCATGC-3’ (*Xho*I, *Sph*I) |
| pMM23-CLZ series constructs | C-R2 | 5’-AAAAGCATGCCTAATCGCCATCTTC-3’ (*Sph*I) |
|  | 1CLZ-F | 5’-GCGCGAATTCATGCTTCATCGTCGGTCC-3’ (*Eco*RI) |
|  | 3CLZ-F | 5’- AAAAGAATTCATGGCCCGCGCT -3’ (*Eco*RI) |
|  | *Max* linker | 5’- GGTACCATGCGAAGGAAAAACCACACACACCAGCAAGATATT  GACGACCTCAAGCGGCAGAATGCTCTTCTGGAGCAGCAAGTCCGTGCACTGGAGGGCGGGGGGGTCTGGGTCTGGGGAATTC -3’ (*Kpn*I, *Eco*RI) |
| pMR1 | Gus-F | 5’-CCGCTCGAGATGGTAGATCTGAGGGTAAATTTCT-3’ (*Xho*I) |
|  | Gus-R | 5’-ACATGCATGCTCACACGTGATGGTGATGGTGATGGC-3’ (*Sph*I) |
|  | lhl-F | 5’- CCCCGGTACCATAACTTCGTATAGCATACATTATACGAAGTTAT  ATGAAAAAGCCTGAACTCACC-3’ (*Kpn*I, *lox*) |
|  | lhlnos-R | 5’-CCCCCTCGAGATAACTTCGTATAATGTATGCTATACGAAGTTAT  CTATTTCTTTGCCCTCGGACGA-3’ (*Xho*I, *lox*) |
| pCambia-N series constructs | Nnls-F | 5’-CCCCAGATCTCCACCATG CCGAAAAAAAAACGCAAAGTG TCCAATTTACTGACCGTACAC-3’(*Bgl*II, Kozak sequence, SV40 NLS) |
|  | Nlz-R | 5’-GGGTTACCTCACGCACAAGAGTTCCGTAGCTGTTC-3’ (*Bst*EII) |
|  | 2N-R2 | 5’-GGGTTACCTAACCAGTGAAACAGC-3’ (*BstE*II) |
|  | 3N-R2 | 5’-GGGTTACCATTTTCTGACCCGGCAA-3’ (*Bst*EII) |
|  | 5N-R2 | 5’-GGGTTACCCTACCCACCGTCAGTACGTGAGATATC-3’ (*Bst*EII) |
| pCambia-C-luc series constructs | CLZnls-F | 5’-GCGCGGTACCCCACCATAACTTCGTATAGCATACATTATACGAAGTTAT ATGCCGAAAAAAAAACGCAAAGTGCGAAGGAAAAACCACACA-3’ (*Kpn*I, Kozak sequence, *lox*, SV40 NLS) |
|  | 1Cnls-F | 5’-GCGCGGTACCCCACCATAACTTCGTATAGCATACATTATACGAAGTTAT ATGCCGAAAAAAAAACGCAAAGTGCTTCATCGTCGGTCC-3’ (*Kpn*I, Kozak sequence, *lox*, SV40 NLS) |
|  | 3Cnls-F: | 5’-AAAAGGTACCCCACCATAACTTCGTATAGCATACATTATACGAAGTTAT ATGCCGAAAAAAAAACGCAAAGTGGCCCGCGCT-3’ (*Kpn*I, Kozak sequence, *lox*, SV40 NLS) |
|  | C-R3 | 5’-GCGCATATGCTAATCGCCATCTTC-3’ (*Nde*I) |
|  | T^ubi1^-F | 5’-CGCCATATGATGGAGCTGCTGCTGTTC-3’ (*Nde*I) |
|  | T^ubi1^-R | 5’-TCGACTGCAGTGCCAAGTGCCAAGTCG-3’ (*Pst*I) |
|  | T^ubi1^-F2 | 5’-TCGACTGCAGATGGAGCTGCTGCTGTTC-3’ (*Pst*I) |
|  | T^ubi1^-lox-R | 5’-CCGGAGATCTATAACTTCGTATAATGTATGCTATACGAAGTTAT  TGCCAAGTGCCAAGTCG-3’ (*Bgl*II, *lox*) |
|  | luc-F | 5’-GAAGATCTATGGAAGACGCCAAAAACATAAA -3’ (*Bgl*II) |
|  | luc-R | 5’ GGGTTACCTTACACGGCGATCTTTCCGCCCTTCTT-3’ (*Bst*EII) |
| pCambia-bar-luc | Bar-F | 5’- GGGGTACCATAACTTCGTATAGCATACATTATACGAAGTTATAT  GAGCCCAGAACGACG-3’(*Kpn*I) |
|  | Bar-R | 5’-GCGCATATGTCAGATCTCGGTGACGGGCAGGACCGGACGGGGCGGTACCGG-3’ (*Nde*I) |
| pCambia-Cre | Nnls-F | 5’-CCCCAGATCTCCACCATG CCGAAAAAAAAACGCAAAGTG TCCAATTTACTGACCGTACAC-3’(*Bgl*II, Kozak sequence, SV40 NLS) |
|  | C-R4 | 5’-GGGTTACCCTAATCGCCATCTTC-3’ (*Bst*EII) |
